# Efficacy of Residual Site Radiation Therapy (ISRT) in Patients with Primary Mediastinal Lymphoma with Deauville Score 4 Following R-CHT: Results of a Retrospective Mono Institutional Study

**DOI:** 10.3390/jcm12113777

**Published:** 2023-05-31

**Authors:** Giuseppe Facondo, Mattia Serio, Gianluca Vullo, Maria Paola Bianchi, Sabrina Pelliccia, Alice Di Rocco, Tiziana Lanzolla, Maurizio Valeriani, Arianna Di Napoli, Agostino Tafuri, Maurizio Martelli, Mattia Falchetto Osti, Vitaliana De Sanctis

**Affiliations:** 1 Department of Medicine and Surgery and Translational Medicine, Sapienza University of Rome, Radiotherapy Oncology, St. Andrea Hospital, 00189 Rome, Italy; 2 Hematology Institute, Sapienza University of Rome, St. Andrea Hospital, 00189 Rome, Italy; 3 Department of Translational and Precision Medicine, Hematology Institute, Sapienza University of Rome, Umberto I, 00189 Rome, Italy; 4 Nuclear Medicine Unit, Department of Medical-Surgical Sciences and Translational Medicine, Sapienza University of Rome, St. Andrea Hospital, 00189 Rome, Italy; 5 Pathology Unit, Department of Clinical and Molecular Medicine, Sapienza University of Rome, St. Andrea Hospital, 00189 Rome, Italy

**Keywords:** lymphoma, primary mediastinal lymphoma, radiotherapy, residual site radiation therapy

## Abstract

Background: In order to evaluate the efficacy of residual site radiation therapy (RSRT) in terms of progression-free survival (PFS) and overall survival (OS) in patients with primary mediastinal lymphoma (PMBCL) with Deauville Score 4 (DS 4) following rituximab and chemotherapy treatment (R-ICHT). Methods: Thirty-one patients with PMBCL were recruited. After completion of R-ICHT, patients were staged with 18F-fluorodeoxyglucose positron-emission tomography, showing DS 4, and were treated with adjuvant RSRT. The chosen techniques for RT delivery were intensity-modulated radiation therapy (IMRT) or three-dimensional conformal RT (3D-CRT). Most patients underwent the first one using cone-beam computed tomography (CBCT). All patients were evaluated every 3 months for the first 2 years and every 6 months afterwards for a period of at least 5 years, with clinical and radiological procedures as required. Results: All patients received RSRT with a dose of 30 Gy in 15 fractions. The median follow-up time of 52.7 months (IQR: 26–64.1 months). The 5-year OS rate was 100%. The 2-year and 5-year PFS rates were 96.7% and 92.5%, respectively. Patients with relapsed disease had been treated with high-dose chemotherapy (HDC) and autologous stem cell transplantation (auto-SCT). Conclusion: RSRT in patients with PMBCL treated with ICHT and DS 4 did not impact unfavorably on patient survival.

## 1. Introduction

Primary mediastinal large B-cell lymphoma (PMBCL) is a rare subtype of non-Hodgkin lymphoma (NHL) that, due to its peculiar clinical and histopathological characteristics, is recognized as a specific entity in the latest World Health Organization classification of lymphoid tumors [1]. Treatment approaches are based on systemic chemo-immunotherapy with or without adjuvant mediastinal radiotherapy. While after DA-R-EPOCH (etoposide, prednisone, vincristine, cyclophosphamide, doxorubicin, and rituximab), adjuvant radiotherapy (RT) was not considered, after R-CHOP/R-MACOP-B, mediastinal radiotherapy has been routinely used in an adjuvant setting in clinical practice [2,3,4]. Interest has increased in evaluating whether post-immunochemotherapy 18-fluorodeoxyglucose (FDG) positron emission tomography computed tomography (PET-CT) can be valuable for guiding subsequent treatment decisions for patients with PMBCL, especially when considering mediastinal adjuvant RT. The National Comprehensive Cancer Network (NCCN) guidelines recommend that PET-CT scans should be interpreted by the 5-point Deauville score (DS) and Lugano response criteria on the basis of visual assessment. This strategy may better stratify patients according to metabolic response, adapting the treatment strategy [5,6]. We are awaiting the data of the IELSG37 randomized trial that investigated the role of adjuvant radiotherapy in patients with PMBCL who obtained a metabolic complete response (Deauville Score 1-3) after R-chemotherapy. In this randomized study, patients with DS 1–3 were randomized to adjuvant radiotherapy versus observation, while patients with DS 4–5 were assigned to best clinical practice according to the choice of the single participating center. Pinnix et al. showed a worse PFS (5-year PFS 62% vs. 100%, *p* = 0.00004) when patients with DS 4–5 after treatment with R-CHOP, R-HCVAD, or R-EPOCH were compared with patients with DS 1–3 [7]. In a series of 156 PMBCL patients treated with DA-R-EPOCH, the EOT PET with DS 5 was correlated with worse OS [8]. This dismal prognosis was also confirmed by Filippi et al. and Vassilakopoulos et al. when stratifying DS 4–5 PMBCL patients receiving adjuvant RT [9,10]. So, the prognosis of patients with DS 4–5 was worse, and the most useful approach was not well defined (RT, second-line chemotherapy, HDC-SCT). As reported in a review by Hoppe et al., in patients with EOT PET DS 4, radiotherapy was routinely offered in clinical practice [11]. To date, there is no unanimous consensus based on strong scientific evidence about the best personalized strategy for only DS 4 patients. In our retrospective series, we analyzed 31 DS 4 patients consecutively treated with residual site radiation therapy (RSRT) in order to assess the local control rate and survival outcomes.

## 2. Materials and Methods

### 2.1. Patients

We retrospectively analyzed 31 patients with PMBCL showing DS 4 at the end of systemic chemo-immunotherapy treated with RSRT between 2010 and 2022 at our institution. The system adopted for classification was the Ann Arbor staging. The criteria for defining bulky disease have been stated as a mediastinal mass greater than one third of the thoracic diameter or a mediastinal mass with a diameter at the widest point of >10 cm. Prior to RSRT, all patients were studied by 18F-fluorodeoxyglucose (FDG) positron emission tomography computed tomography (PET/CT) for assessment and restaging of response to treatment according to the Deauville scoring system. PET-TC was done at two institutions by nuclear medicine specialists who are experts in the field of lymphoma. All the PET scans were centrally reviewed by two expert nuclear medicine specialists.

The decision for RT was made based on consensus at institutional multidisciplinary board meetings in the presence of hematologists, taking into account the bulky disease, number of chemotherapy cycles, and response to chemotherapy. All patients signed informed consent before starting treatment.

### 2.2. Chemotherapy

All patients had been treated with rituximab-containing regimens, either R-MACOP (12 weeks) or R-CHOP (6 cycles every 21 days). The total number of rituximab infusions was 8 in all different schemes. Five patients underwent autologous stem cell transplant (auto-SCT) as early intensification, and two patients underwent a second line of systemic therapy for relapsed disease.

### 2.3. Radiation Therapy

The immobilization systems used were the wing board with raised arms or the thermoplastic head-neck mask. A planning non-contrast CT scan was taken with 25 mm slices. By using PET-CT and/or CT with i.v. contrast, the residual mediastinal mass after chemotherapy was identified and, through precise fusion procedures, contoured as gross tumor volume (GTV) by two experienced radiation oncologists (VDS and MFO). The planning target volume (PTV) was created by an isotropic expansion of 5 mm from the GTV, taking into consideration surrounding healthy structures. RSRT was administered by conventional fractionation (fractions per day: 2.0 Gy) five days per week by a 6-MV linear accelerator for a total dose of 30 Gy.

**Figure 1:**
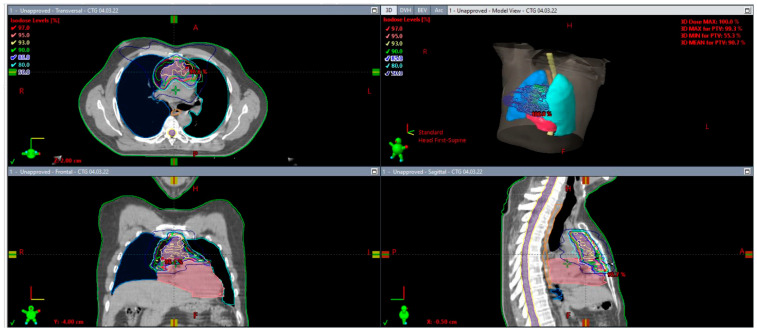
Involved site radiation therapy with the Eclipse 4.5.5 (Varian) treatment planning system.

The techniques chosen for RT delivery were intensity-modulated radiotherapy (IMRT) in 28 patients and three-dimensional conformal radiotherapy (3D-CRT) in 3 patients only. All along the RT course, the portal vision was planned every 2 days for patients treated with 3DCRT. Daily on-board imaging with cone-CT was planned for patients treated with the IMRT technique. No deep inspiration breath-hold technique was adopted in our series of patients. The Eclipse 4.5.5 (Varian) treatment planning system was used for all patients’ radiotherapy plans. Details of target and organ at-risk delineation and an example plan are shown in Figure 1.

### 2.4. Follow-Up

Patients were followed up with PET-CT or contrast-enhanced CT scans 3 months after the completion of RT. The complete responders were reviewed every 3 months for the first 2 years, alternating with the hematologist, and every 6 months from the 3rd to the 5th year. A full blood count and lactate dehydrogenase were checked at each follow-up. Toxicities were evaluated according to the Radiation Therapy Oncology Group (RTOG) scale for acute and late adverse effects at each follow-up [12].

### 2.5. Statistical Analysis

Overall survival (OS) was defined as the time from diagnosis to death due to any cause. Progression-free survival (PFS) was calculated from the date of diagnosis to progression, relapse, death due to any cause, or the last follow-up. The Kaplan-Meier method was used to estimate the rates of survival analysis, and statistical differences were evaluated with the log-rank test. Statistical analysis was performed using the SPSS statistical software package version 25.0 (SPSS, Chicago, IL, USA).

## 3. Results

The characteristics of the 31 patients evaluated are listed in Table 1. The median age at diagnosis was 34 years (IQR 28–44 years). All patients had bulky mediastinal disease. Eighteen (58%) patients were male, and 13 (41.9%) were female. Four (12.9%) patients had stage I, 25 (80.6%) had stage II, and only two patients (6.4%) had stage IV before treatments. All the patients showed DS 4 on PET-CT at the end of systemic therapy.

All patients were treated with standard ICHT: 11 patients (35.4%) had received R-CHOP (rituximab, cyclophosphamide, doxorubicin, vincristine, and prednisolone); R-MACOP-B (rituximab, methotrexate, doxorubicin, cyclophosphamide, vincristine, prednisone, and bleomycin) was given to 20 patients (64.5%). Five patients underwent high-dose chemotherapy (HDC) and an autologous stem cell transplant (auto-SCT) as early intensification for DS 4 after I-line chemotherapy. 

The median time to the auto-SCT from the end of ICHT was 19.7 weeks (IQR: 16.1–37.3) for 5 patients who underwent early intensification. In all five patients, the PET after auto-SCT showed a DS 4.

All 31 patients underwent mediastinal adjuvant RSRT after a median time from the end of ICHT or auto-SCT of 9 weeks (IQR 8.2–14.7).

The median total RT dose was 30 Gy (range 30–40 Gy). The median CTV was 110.3 cc (IQR 77.5–208.1 cc), and the median cc of the PTV was 266.1 cc (IQR 219.1–421 cc). Three (9.6%) patients were treated with conventional 3D-CRT and 28 (90.6%) patients with the IMRT technique.

In all patients, PET-CT was repeated after a median of 13.4 weeks (IQR: 12.8–14.5) from the end of RSRT. The DS after RSRT was recorded as follows: 4 patients had a complete response (DS 1–3); 27 patients had a partial response at the PET-TC (DS 4). At a median follow-up time of 52.7 months (IQR: 26–64.1 months), the 5-year OS rate for the entire group was 100%.

**Figure 2:**
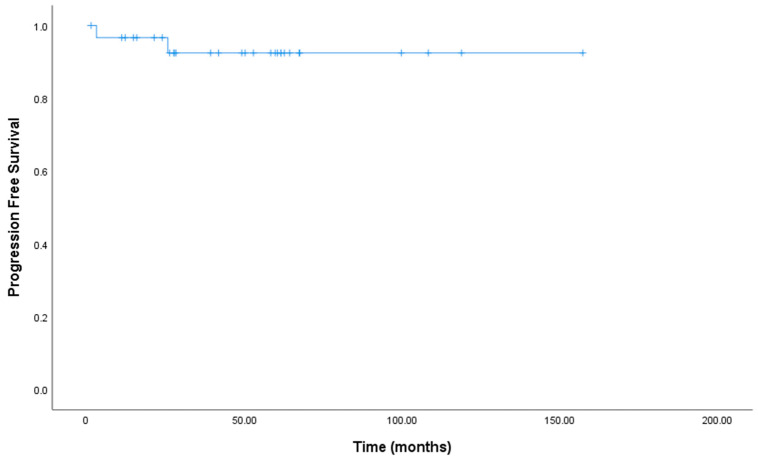
Kaplan–Meier curves for Progression Free Survival in all of 31 patients.

The 2-year and 5-year PFS rates were 96.7% and 92.5%, respectively (Figure 2). The median OS and PFS times have not been reached.

**Figure 3:**
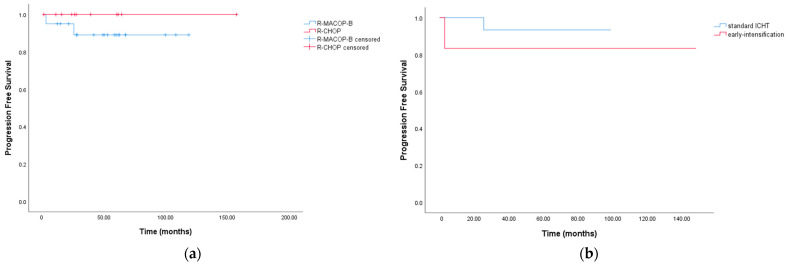
(**a**) Kaplan–Meier curves for Progression Free Survival between patients who underwent R-CHOP or R-MACOP-B treatment; (**b**) Kaplan–Meier curves for Progression Free Survival between patients who underwent standard ICHT vs. early-intensification.

Two patients experienced disease relapse at 9 and 25.5 months, respectively, after radiation treatment and received high-dose chemotherapy (HDC) and an autologous stem cell transplant (auto-SCT). No statistically significant differences were found in 5-year PFS among patients in the two ICHT groups (*p*-value = 0.33) (Figure 3a), nor between patients who underwent early intensification versus patients treated with only standard chemotherapy (*p*-value = 0.32) (Figure 3b).

In terms of toxicities, only 5 patients (16.1%) experienced acute G1-G2 toxicities. Of these patients, two developed G1 asthenia, one developed G2 neutropenia, and two developed G1 nausea. Nutritional supplements were prescribed to patients who showed asthenia, and only the patient with nausea was subjected to antiemetic drugs. Every acute toxicity reported was well treatable. Resolution of all acute symptoms occurred after a short time. Three patients showed late G1-G3 toxicities. One patient experienced a G2 pulmonary infection that resolved with one course of antibiotics. Other patients had G1 and G3 neutropenia. Table 2 shows the toxicology characteristics of all patients. 

## 4. Discussion

PET-CT imaging plays a central role in choosing the right therapeutic approach for PMBLC patients [13,14]. In particular, the PET-CT Deauville score has been shown by multiple studies to be a valuable tool to guide tailored subsequent treatment and to predict survival rate after ICHT for PMBCL [15,16,17].

Incorporating PET/CT imaging into RT planning has shown several positive effects, such as improved identification of vital lymphoma tissue, leading to field expansion or reduction. The concepts of involved node radiation therapy (INRT) have been introduced to spare normal tissue by using smaller treatment fields with minimal safety margins. Modern treatment volume concepts rely on PET scans fused with planning CT scans or scans performed in the treatment position. ISRT is now the international standard of care, and implementing these concepts in clinical practice remains challenging and requires PET/CT imaging for interpreting and defining treatment volumes [18].

In 2021, the American Radium Society Appropriate Use Criteria published a comprehensive systematic review and provided evidence-based guidelines regarding post-ICHT PET-based RT strategies for PMBCL patients. Among the 72 DS-4 patients included in this review, all were treated with radiotherapy, and 13 relapses were recorded. Considering the lack of prospective trials omitting adjuvant RT, panel experts strongly recommend consolidative RT when R-chemotherapy is administered. When ISRT is used, doses ranging from 36 to 40 Gy are considered appropriate, with a possible boost up to 40 to 50 Gy directed to the residual mediastinal disease only [11].

Belinda et al. analyzed the scientific evidence in the PET era, which could lead to tailored and personalized consolidative radiotherapy rather than omitting it. When focusing on the role of RT in PET-positive patients after immunochemotherapy, the authors concluded that RT has an emerging and potential role in converting incomplete responses. Considering the improved outcomes with only limited severe toxicities (mainly radiation dermatitis G3–4), they support the use of RT as a non-cross-resistant second-line treatment strategy [19].

Also, the latest 2023 German evidence-based guideline on diffuse large B-cell lymphoma discusses RT’s role in depth. Lacking robust prospective data, according to the authors, the mainstay of consolidative therapy for residual DS 4–5 patients after six cycles of R-CHOP is still modern ISRT, which allows for similar outcomes in this cohort compared to PET-negative patients. Treatment planning should be implemented by PET-CT co-registration to better discriminate the vital residual tissue (GTV) and ensure an adequate CTV, which should include macroscopic post-chemotherapy tumor tissue. Doses between 36 and 40 Gy in 2 Gy per fraction are suggested as a standard, regardless of the RT techniques [20].

Many studies in the literature incorporate DS 4–5 and naturally have worse results than DS 1–3. The Korean Radiation Oncology Group analyzed 512 patients sorted into two arms: the DS 4–5 arm (n = 24) was matched at a 1:2 ratio with the DS 1–3 arm (n = 48) using the propensity score matching method. The 5-year locoregional recurrence-free survival rates were 88.8% in the DS 1–3 arm and 74.3% in the DS 4–5 arm, respectively (*p* = 0.155). The 5-year distant failure-free survival rates were 91.1% in the DS 1–3 arm and 84.3% in the DS 4–5 arm, respectively (*p* = 0.333). The five-year recurrence-free survival rates for the DS 1–3 arm and the DS 4–5 arm were 86.6% and 66.8%, respectively [21].

As shown by a study from Martelli et al., DS 1 to DS 3 post-R-chemotherapy identified patients with good outcomes, while DS 4 to DS 5 was associated with worse survival, in particular patients with DS 5. In fact, their study reported progressive disease in seven patients (DS 4–5) who received autologous stem cell transplantation or underwent second-line chemotherapy, obtaining complete remission in all but two. The other 27 DS 4–5 patients received radiation therapy, becoming DS 1–2 on 11/27 (48%). For the remaining 12 patients, they recorded 3 with DS 3, 5 with DS 4, and 4 with DS 5; only 3 patients showed progressive disease, all with DS 5. The authors concluded that patients with DS 3 can be considered at low risk of failure, unlike patients with DS 4–5. In this paper, patients with DS 4 and DS 5 were analyzed together, showing a 5-year PFS and OS of 68% and 83%, respectively, although the true dismal prognosis seems to be associated with DS 5 [22]. 

In 2013, Filippi et al. reported the outcomes of a cohort of 37 PMBLC patients treated with mediastinal RT stratified by DS response. All 14 DS 4 patients obtained CR, as did other patients with DS 3. Conversely, DS 5 patients experienced significantly worse outcomes. Out of 4 patients with a score of 5, 1 showed complete response (25%), 2 had perpetual positivity (50%), and 1 presented progressive disease (25%) [23]. A subsequent study from the same authors analyzed a series of combined treatments in 51 patients with PMBCL and obtained similar results. In particular, results showed that DS 4 patients receiving RSRT had the same outcomes as DS 3 patients, with a good long-term prognosis and no recurrence [9].

The standard therapeutic approach for all PMBCL in our institute is represented by a combination of R-CHOP or R-MACOP-B as first-line chemotherapy. ISRT may represent an advantageous and safe approach in all those patients with PMBCL who did not have a complete metabolic upshot after ICHT/auto-SCT (DS 4–5). Therefore, at our institution, all DS 4 patients are treated with ISRT.

In our retrospective study of 31 patients, we show that patients with DS 4 who received ISRT after ICHT or auto-SCT regimens had excellent outcomes with limited acute toxicity. Among the analyzed cohort, only two patients (6.45%) experienced disease relapse after immunochemotherapy and RT. For those relapsing patients, however, multimodality salvage therapy with additional high-dose chemotherapy and autologous stem cell transplantation was effective. thus globally resulting in highly favorable 5-year OS rates of 100% for patients included in the study. The outcomes of the whole analyzed population appear comparable with those reported by other studies in the literature [24,25,26,27,28].

Evaluating exclusively patients with DS 4, demonstrating an excellent result both in terms of survival and toxicity, was the goal of this study. The efficacy of RT in disease control in most patients with residual 4 DS after R-CT is highlighted by these results, which also confirm the outcomes of other previously published series founded on different functional imaging evaluations at the end of chemotherapy in the era before rituximab. The retrospective nature of the study and the limited number of patients included, resulting in a small number of events, represent a limitation of this study. A strong point of this study is to have all the PET-CTs reviewed by two different nuclear medicine specialists at our institution, thus strengthening the DS 4 reports. Naturally, in the absence of a biopsy confirming the histology of PET findings, the bias of overestimating the DS 4 and creating false DS 4 remains.

## 5. Conclusions

In this patient cohort, we analyzed the toxicity profile, OS, and PFS of patients with PMBCL DS 4 treated with ICHT and RSRT. Patients have shown excellent results in terms of survival and profile of toxicity. Our study should prompt larger prospective and multi-institutional series to better confirm the role and advantage of this RT approach in PMBCL.

## Figures and Tables

**Table 1:** Patients and radiation therapy characteristics of all 31 patients.

Characteristic N: 31 Patients	n (%) Median (IQR)
Age (year)	34 (28–44)
*Sex*	
Male	18 (58)
Female	13 (41.9)
*Stage*	
I	4 (12.9)
II	25 (80.6)
IV	2 (6.4)
*Chemotherapy*	
R-CHOP	11 (35.4)
R-MACOP-B	20 (64.5)
Duration CHT (week)	12 (10.5–13.8)
T CHT-RT (week)	9 (8.2–14.7)
Total dose (Gy)	30
Fractions (n)	15
Dose per fraction (Gy)	2
*Planning technique*	
3D-CRT	3 (9.6)
IMRT	28 (90.3)
CTV, (cc)	110.3 (77.5–208.1)
PTV, (cc)	266.1 (219.1–421)

CTV: clinical tumor volume; 3D-CRT: three-dimensional conformal radiotherapy IMRT: intensity-modulated radiotherapy; PTV: planning tumor volume.

**Table 2:** Acute and late toxicities of all patients.

Toxicities 31 Patients	N (%)
Acute toxicities	5 (16.1)
Nausea G1	2
Asthenia G1	2
Neutropenia G2	1
Late toxicities	5 (16.1)
Neutropenia G1	2
Neutropenia G3	1
Pulmonary infection G2	1

## Data Availability

Not applicable.
